# Effect of Aerobic Exercise versus Non-Invasive Brain Stimulation on Cognitive Function in Multiple Sclerosis: A Systematic Review and Meta-Analysis

**DOI:** 10.3390/brainsci14080771

**Published:** 2024-07-30

**Authors:** Mahmoud Elkhooly, Arianna Di Stadio, Evanthia Bernitsas

**Affiliations:** 1 Department of Neurology, Southern Illinois University School of Medicine, Springfield, IL 62702, USA; elkhoolymahmoud27@outlook.com; 2 Department of GF Ingrassia, University of Catania, 95121 Catania, Italy; 3 IRCSS Santa Lucia, 00179 Rome, Italy; 4 Department of Neurology, Wayne State University, Detroit, MI 48201, USA

**Keywords:** multiple sclerosis, noninvasive brain stimulation, cognitive impairment, aerobic exercise

## Abstract

Objective: In this study, we investigated the effects of noninvasive brain stimulation (NIBS) and exercise on cognition in patients with multiple sclerosis (pwMS). Methods: A literature search was performed using the Cochrane Library, Scopus, PubMed and Web of Science. The time interval used for database construction was up to February 2024; the collected trials were subsequently screened, and the data were extracted. Results: We identified 12 studies with 208 pwMS treated with noninvasive brain stimulation. Seven of the twelve studies concluded that NIBS was effective in improving reaction time, attention and processing speed. Additionally, 26 articles investigated the effect of various types of exercise on cognition among 708 pwMS. Twelve studies used aerobic exercise only, three studies used resistance only, one used yoga, and ten studies used mixed forms of exercise, such as Pilates, resistance and Frenkel coordination. Aerobic exercise was effective in improving at least one cognitive domain in ten studies. Resistance exercise was found to improve cognition in three studies. Yoga failed to show any improvement in one study. Conclusions: NIBS might be an effective intervention for cognition improvement among pwMS. Aerobic exercise and combined forms of exercise are the most frequently investigated and applied and found to be effective. Further studies are needed, especially for resistance, balance and stretching exercises.

## 1. Introduction

Multiple sclerosis (MS) is an autoimmune, chronic, demyelinating, inflammatory disease of the central nervous system that is characterized by demyelination and axonal degeneration [1]. MS is classified as primary progressive, secondary progressive or relapsing–remitting [2]. MS prevalence frequently demonstrates a trend of rapid global expansion, with the highest prevalence in North America and Europe [3,4]. It is typically characterized by a range of clinical presentations, including cognitive impairment (attention deficit and executive function), motor impairments, sensory abnormalities and visual impairments (e.g., optic nerve dysfunction) [5]. 

Cognitive impairment is common in MS and impacts 45–70% of patients of people with MS (pwMS) [6]. It may manifest early in the disease and deteriorate as the disease progresses, impairing work performance and worsening quality of life [7]. MS–cognitive impairment can be multifactorial, mainly related to cortical and subcortical pathology with a possible contribution from the white matter [8,9]. Currently, there is no FDA-approved treatment to stabilize cognitive decline and clinicians are using different, non-pharmacological ways to manage cognitive dysfunction in pwMS [10]. Disease-modifying therapies (DMTs) decrease the frequency of MS flare-ups and delay the disease’s natural progression. Therefore, DMT administration may have a positive effect on fatigue and cognition [11,12]. Nevertheless, a subset of pwMS may encounter adverse effects, prompting the exploration of supplementary and alternative therapies with the aim of achieving a harmonious equilibrium between the effectiveness and safety of the medication [13]. Moreover, the role of education and cognitive enrichment may serve as a cognitive reserve and prevent or stall cognitive decline [14]. MS symptoms, such as fatigue, depression and sleep deprivation, can negatively impact cognition and should be promptly addressed. 

Recently, noninvasive brain stimulation (NIBS) has garnered considerable scholarly interest [15] as a supplementary therapeutic approach for neurological disorders. NIBS is generally recognized for its ability to noninvasively induce excitatory changes in the underlying cerebral cortex via the application of electromagnetic energy. Furthermore, it can potentially induce long-lasting neuroplasticity changes [16]. The most frequently used NIBS techniques in clinical settings are repetitive transcranial magnetic stimulation (rTMS) and transcranial direct current stimulation (tDCS) [17,18]. To date, researchers have experimentally explored the effects of the NIBS technology on the cognitive function of pwMS. However, because of the different designs, the experimental results remain controversial, to some extent. This necessitates a more rigorous investigation into the impacts of NIBS on MS. 

There are few behavioral interventions to manage cognitive impairment with exercise training and cognitive rehabilitation. There is growing evidence regarding the impact of exercise on cognition among pwMS, with conflicting results. Ozkul et al. [19] discovered a link between improvements in mood, quality of life and cognition following exercise, as well as the positive benefits of combining aerobic and Pilates exercise on cognitively impaired pwMS. Furthermore, Sandroff et al. [20,21,22] demonstrated that exercise is a potentially useful treatment for MS-related cognitive impairment. However, Savšek et al. reported improved walking speed and brain-derived neurotrophic factor (BDNF) levels after aerobic exercise but did not demonstrate a positive effect on cognitive function [22]. According to Langeskov-Christensen et al., 24 weeks of supervised progressive aerobic exercise did not affect MS patients’ cognitive function [23]. Additionally, Kooshiar et al. [24] demonstrated the benefit of aquatic exercise on fatigue alleviation and quality of life in pwMS but not on cognitive performance.

As no consensus between experts has been reached and results on the role of NIBS and exercise are still non-conclusive, we performed a systematic review to present data from clinical randomized trials on the effects of current NIBS techniques and different forms of exercise on MS-related cognitive impairment. 

## 2. Methods

**Figure 1:**
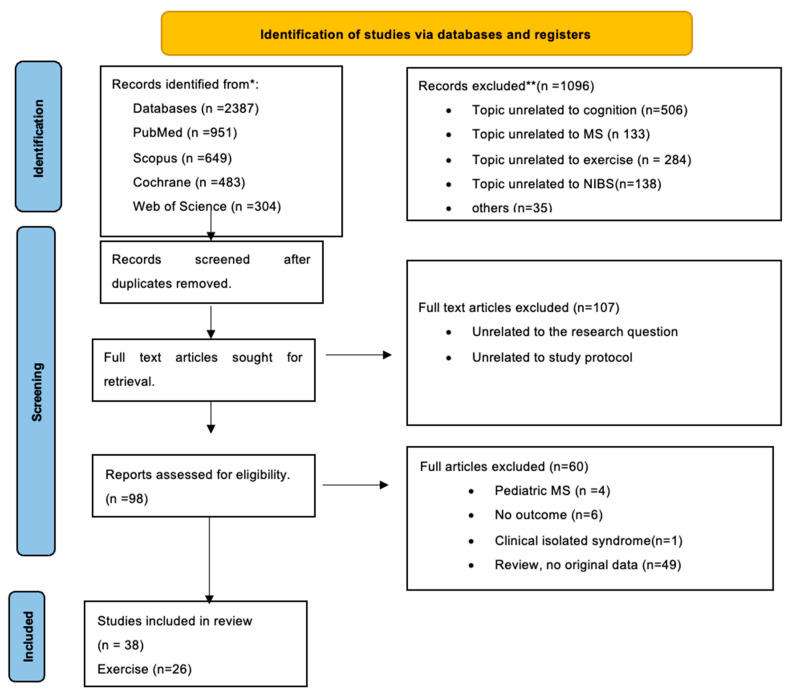
Flow diagram of identification process for eligible studies per PRISMA guidelines. *All the identified records from different databases. ** All the excluded records including topics unrelated to cognition, MS, Exercise, non-invasive brain stimulation and others (as not written in English).

In accordance with the Preferred Reporting Items for Systematic Reviews and Meta-Analyses (PRISMA) recommendations, a comprehensive and systematic literature search was conducted by two reviewers (ME and EB) that was resolved through discussion among the reviewers until agreement was achieved. We included papers from January 2004 to February 2024. This systematic review adheres to the PRISMA guidelines (http://www.prisma-statment.org (accessed on 10 July 2024)) and has PRISMA registration number 567,064. The review’s characteristics did not necessitate approval from an Institutional Review Board. Four distinct electronic databases (PubMed, Scopus, Cochrane, and Web of Science) were searched [25], as shown in Figure 1. The protocols outlined by the NHS Centre for Reviews and Dissemination were strictly followed [26].

The subsequent word combinations were employed in our search: “cognition”, “exercise”, “noninvasive brain stimulation, “stabilization”, “balance”, “core”, “aerobic”, “Symbol digit modality test”, “stretching”, “resistance”, “Yoga”, “PASAT” and “memory impairment.” Furthermore, a comprehensive search was conducted on ClinicalTrials.gov, a database operated by the National Institutes of Health, to locate related registered and ongoing trials. The search was conducted through February 2024 and included studies published since 2004. Articles written in a language other than English were excluded. Additionally, we excluded any studies not related to the research question. 

To maintain the standard of this evaluation, precise inclusion and exclusion criteria were implemented. Per the inclusion criteria, pwMS must have a confirmed diagnosis of MS according to the 2005, 2010 and 2017 McDonald criteria, being older than 18 years old, being exposed to NIBS, and engaged in exercise with an outcome evaluated using diverse cognitive assessment modalities [27,28,29]. The exclusion criteria included studies not pertaining to exercise or NIBS, studies with subjects younger than 18 years old, and those lacking a certain outcome. Once eligible studies were identified, the subsequent information was extracted from each article: authors’ names and title, year of the study, sample size and study design, type, frequency and duration of each intervention, main outcome and outcome metrics. The risk of bias in the included studies was analyzed using Robvis software for bias assessment [30]. The assessment categories consist of random sequence generation, allocation concealment, blinding of participants and personnel, blinding of outcome assessment, inadequate outcome data, selective reporting and other biases.

### Data Extraction and Statistical Analyses

We extracted the following data—when available—to perform statistical analyses: improvement, method used to examine the cognitive function and numeric scores of cognitive tests before and after treatment. Nominal data were analyzed using Fisher (F) test whether numeric data were analyzed via one-way ANOVA. *p*-value was considered significant <0.05. PRISM 10 was used to perform statistical analyses.

## 3. Results

A total of thirty-eight studies with 1641 participants (310 i in NIBS group; 1331 in exercise group) were included. A total of 916 patients were analyzed as an intervention group (708 in the exercise and 208 in NIBS). We included 725 participants as a control in both the NIBS and exercise groups. In most studies, no data about the extent of the cognitive impairment in the control/intervention group were found. The control group in the NIBS received either sham tDCS or cognitive training (CT). The control participants in the exercise group received either no intervention, regular physical therapy, relaxation exercise or non-structured home-based exercise. 

Various tools to screen for cognitive function were used; fifteen studies used Symbol–Digit Modalities Test (SDMT), twelve used the Paced Auditory Serial Attention Test (PASAT), five used California Verbal Learning (CVLT), five used the Brief Visual Memory Test (BVMT), five used Multiple Sclerosis Quality of Life-54 (MSQOL-54) and four used the Modified Fatigue Impact Scale (MFIS). Some studies used other tools such as Selective Reminding Test (SRT), Word List Generation (WLG), Stroop Color–Word Interference (SCWT), Brief International Cognitive Assessment in MS (BICAMS), Spatial Recall Test (SPART), Regensburg Verbal Fluency Test (RWT), Basic and complex attention (ANT), Test battery for Attention (TAP), Verbal Learning Memory Test (VLMT), Trail Making Test (TMT) and Reaction Time (RT).

Because of the lack of homogeneous methodology to evaluate mental cognition and the presence of numeric scores in four (33.3%) of twelve NIBS studies and eight (30.7%) of the twenty-six exercise studies that would negative impact on the analyses of data creating a bias, we only used improvement or non-improvement as parameter to use in the statical analyses.

### 3.1. Effect of NIBS on Cognition among pwMS

Twelve studies with 310 participants (208 in the intervention group and 102 in the control group) were included. Six crossover studies and six parallel (each participant remains in the assigned group until the end of the study) were performed. Overall, 83% of studies used transcranial direct current stimulation (tCDS), and 8.3% of studies used transcranial random noise stimulation (tRANS) and transcranial alternating current stimulation (tACS). The intensity of the NIBS ranged from 1 to 2 mA. Electrode placement is usually carried out according to the international 10-20 system for EEG electrodes [31]. The selection of the electrode location depends on the brain area involved in the cognitive function of interest, for example, the prefrontal cortex, which controls most higher cognitive functions [32]. Moreover, the function of interest might be lateralized; that is, one of the hemispheres mainly supports it. In this scenario, the actively stimulating electrode is positioned on the left (F3) or right (F4) prefrontal cortex. A central arrangement of the active electrode is possible if no lateralization is anticipated (Fz) [33]. The location of the secondary “reference” electrode is more critical for tDCS than for tACS or tRNS, as a direct current is more sensitive to current flow direction, which results in distinguishing excitatory and inhibitory (anodal and cathodal stimulation) effects, respectively [34,35]. In our review, a variable electrode location was found. Anodes were placed in F3 (nine studies) and C3 (two studies), and one study used P4. Cathodes were placed in different locations, including the right shoulder (three studies), supraorbital (two studies), and F4 (two studies), and one study used each of the other locations, such as the forehead: FP2, CZ and AF4.

In 58% of the studies (7/12), improvement was reported. After analysis of the cognitive domains, attention was improved in 25%, while executive function, speed and cognitive fatigue improved in 16.6% of studies.

In this cohort, SDMT was the most frequently used tool in five studies, followed by basic and complex attention (ANT) and reaction time (RT) (two studies each). Table 1 presents essential study information, including the type of intervention and outcome. 

### 3.2. Effect of Exercise on Cognition among pwMS

Twenty-six studies with 1331 participants were included (708 as an intervention group and 623 as a control group). In most studies, no data were provided on the cognitive status of the participants in the control group. Twelve studies used aerobic exercise alone, one study used yoga, three used resistance exercise and ten used mixed types, such as Pilates and strength exercises. SDMTs were the most frequently used tool, even in combination with other tools (twelve studies), followed by the PASAT-3 test (ten studies), MSQOL-54 (four studies) and MFIS (four studies). Others (nine studies) used different scales, either alone or in combination with other tools for cognitive evaluation, such as CVLT, BVMT, SRT, WLG, SCWT, BICAMS, SPART, RWT, TUG, TAP, VLMT and TMT. Ten studies yielded cognitive memory data measured by at least one of the five different methods: BVMT-R (five studies), CVLT (five studies), SRT (three studies), SPART (three studies) and VLMT (one study). Data on the processing speed were obtained from 17 studies, which were assessed using SDMT, PASAT, IC-RT, DSST and TAP. Furthermore, data on executive function were reported in nine studies, which were evaluated using WLG (three studies), DKFFS (one study), TMT (one study), RWT (one study), MFT (one study), TUG (one study) and SCWT (one study).

Among the twelve studies that used aerobic exercise, eight of them (66%) showed an improvement in cognition when using a variety of tools (PASAT, SPART, SRT, DSST and SDMT), while resistance exercise was used in three studies, and all of them (100%) showed improvement when using MSQOL, MFSC and PASAT tests. Core stabilization was used in one study and was effective in improving cognition when using CVLT, BVMT-R and SDMT. Yoga was used in only one study and failed to show any improvement. Mixed exercises were used in 10 studies, and 90% of them showed an improvement in at least one cognitive domain. 

Table 2 summarizes the effect of different types of exercise on cognition among pwMS.

### 3.3. Risk of Bias Assessment

**Figure 2:**
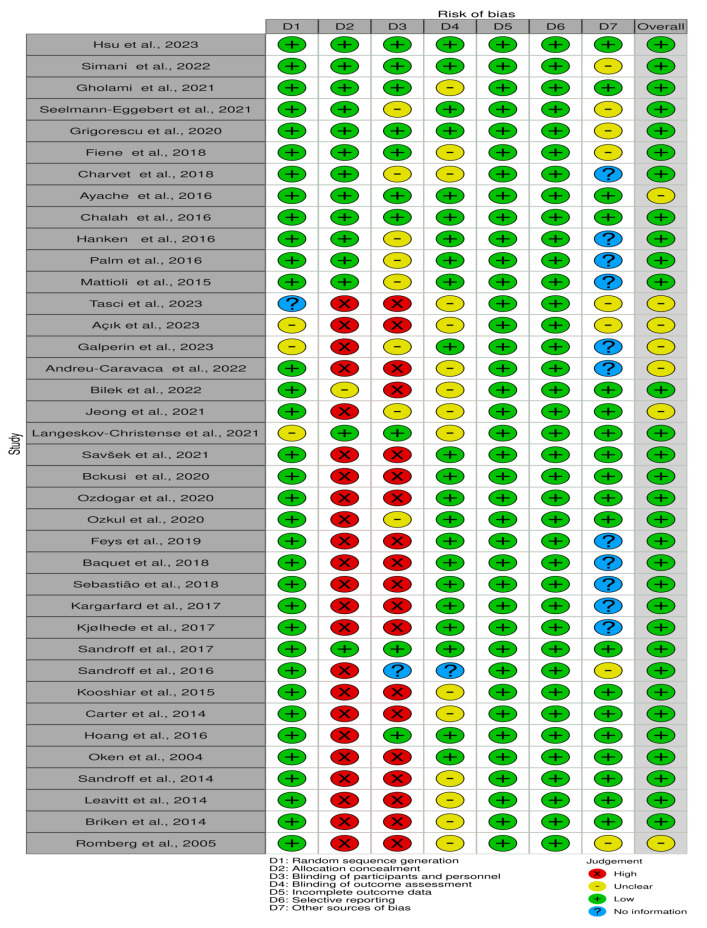
Risk of bias in the included studies [19,21,24,36,37,38,39,40,41,42,43,44,45,46,47,48,49,50,51,52,53,54,55,56,57,58,59,60,61,62,63,64,65,66,67,68,69,70].

Figure 2 outlines the evaluation of bias risk for every study included. Most studies had a low risk of bias; however, the allocation concealment and the blinding of participants and personnel were common challenges. Out of the thirty-eight studies, twenty-three showed a high risk of bias in allocation concealment, and nineteen studies showed a high risk of bias in participant and study staff blinding. 

#### Comparison between NIBS and Exercise on Cognition

**Figure 3:**
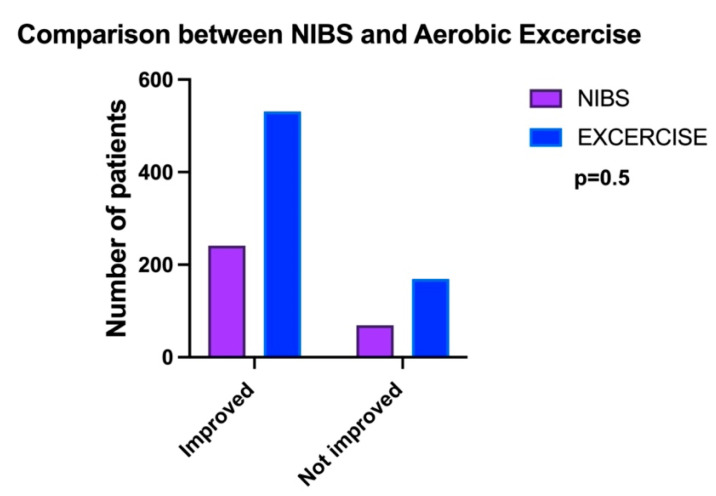
The comparison between NIBS (noninvasive brain stimulation) and aerobic exercise shows no statistically significant differences between the two treatments. To note, there was a statistically significant difference between the number of the patients allocated in the two groups: 310 patients in NIBS and 708 in the exercise.

We did not identify any statistically significant difference between NIBS and exercise (F: *p* = 0.5) (Figure 3) when comparing the patients who recovered and those who did not recover. 

**Figure 4:**
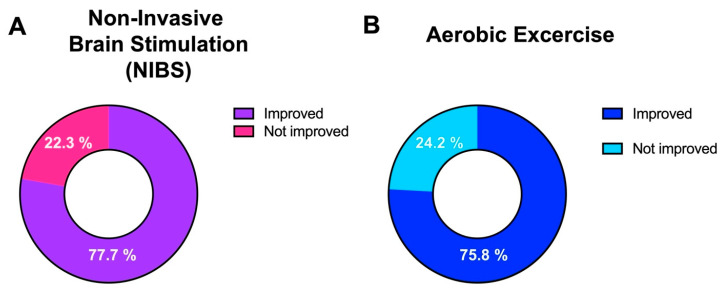
(**A**,**B**) Looking at the prevalence of improvement in the two groups, it appears clear that both NIBS and aerobic exercise offer an amelioration of the cognitive function in patients with MS.

In fact, looking at the likelihood of recovery using the treatment, 77.7% of the sample improved their cognitive skills using NIBS (Figure 4A), and 75.8% of patients who performed aerobic exercise independently from the exercise they did (Figure 4B). 

## 4. Discussion

Overall, the results of our study showed that both NIBS and aerobic exercise can benefit cognition in MS patients. We included a total of 38 English-language papers that investigated 1641 pwMS. Out of the twelve studies that examined the effects of NIBS, seven studies that included 241 participants reported a NIBS-associated improvement in cognition versus five studies that included 69 participants that showed no benefit. Factors that predispose pwMS to respond in a positive way and improve have not been established. Age, disability and comorbidities might play a role in cognitive improvement after NIBS. Li et al. concluded that pwMS younger than 45 years old and those with EDSS scores below 3.5 benefit more from NIBS in terms of improved motor function than older, more disabled patients. In other words, NIBS might be more efficient in younger patients with less functional impairment [6]. Our results showed that NIBS might have positive effects mainly on attention, with limited effects on executive functions and processing speed. 

There is an ongoing debate on the mechanism of NIBS related to cognitive improvement, with the most likely explanation being related to changes in neuroplasticity [71] or cortical excitability [72]. The mechanisms through which different types of NIBS can affect neurological function vary amongst cognitive neuroscience models. For example, transcranial electric stimulation (tES) can induce subthreshold polarization of neurons in the stimulated area, whereas transcranial magnetic stimulation (TMS) can induce suprathreshold depolarization of the neurons [73]. Most studies in our review applied tCDS as an intervention technique. 

Ten out of the seventeen studies examining the effect of exercise on processing speed showed improvement. The exercise was mostly aerobic, performed two to three times a week for a duration from six to twenty-four weeks. Despite the relatively small number of studies where resistance training was applied, it was found to be entirely successful in improving cognition. Our results are in line with previous observations by Li’ et al. and support the overall positive impact of exercise on cognition among pwMS. [74]. On the contrary, Gharakhanlou et al. reported [75] that exercise training had no appreciable impact on global memory or any of the cognitive subdomains. Executive functions have been examined in nine studies, with four of them (44%) showing improvements using a variety of tools (WLG, TUG and TMT). It seems that using a combination of different exercise types has the best outcome rather than using a single intervention. 

Different mechanisms have been proposed to explain the effect of exercise on cognition, such as stimulation of the cellular and molecular processes of angiogenesis, neurogenesis and synaptogenesis [76]. It was reported that exercise can increase the levels of BDNF, which might offer neuroprotection. Moreover, a previous study showed that individuals with diminished BDNF expression in the hippocampus and temporal cortex are more susceptible to neurodegenerative disorders. Additionally, BDNF levels can rise two to three times following acute exercise in comparison to controls, and this increase is positively correlated with enhanced cognitive function [77,78].

Our findings support a moderate effect of aerobic exercise and a more favorable effect of resistance training on cognition. Although most MS experts do not incorporate exercise into their MS treatment regimens, it is now accepted that pwMS may benefit from regular exercise as a means of improving their cognitive function and wellbeing [19,22,60,70,79]. Despite the lack of many details supporting such interventions in MS, it is acknowledged that there is an inverse relationship between the intensity of exercise and the degree of cognitive decline [80].

On a longitudinal basis, exercise is an effective way to prevent and improve cognitive deterioration because, as demonstrated by De la Rosa et al. [81], it can positively impact cognitive function over an extended period of time by improving peripheral redox regulation, delaying physiological memory loss and increasing associated neurotrophy.

Interestingly, both NIBS and exercise showed equally positive results, with no significant difference between them being found. Subsequently, our data do not support the use of one over the other in terms of improving cognitive outcomes. Exercise promotes the anti-inflammatory state in pwMS, and different NIBS techniques may decrease neuroinflammation in MS and stroke. Given that neuroinflammation could contribute to cognitive dysfunction in pwMS [82,83], the results are not surprising [84], and both modalities can be used based on individual characteristics and preferences to prevent cognitive worsening in pwMS.

## 5. Limitations

It is necessary to acknowledge the limitations of this study. First, the exercise interventions were not blinded, which could introduce bias, as our trials were RCTs. Second, various studies have utilized different kinds of cognitive assessment tools, which can occasionally provide inconsistent improvements. Third, we were unable to comprehend the effects of varying exercise intensities on cognitive performance in pwMS since many of the included studies did not describe the intensity of the exercise intervention. Fourth, RRMS was the most prevalent type in our review, while progressive forms of MS were not studied that extensively. Further, several studies only reported the improvement without specifying the magnitude of it; for this reason, we were obliged to consider only nominal data and not numeric values. Obviously, this limitation reduced the power of our statistical analyses. Fifth, we could not evaluate the effect of different disease-modifying therapies on cognitive outcomes. Furthermore, most studies did not include the actual cognitive status of the control group, thus interfering with comparison and data accuracy. Finally, it is important to consider that the study on physical activity has a series of cofounders that might impact our results, i.e., variable exercise protocols; therefore, this is something to keep in mind when we interpret our findings.

## 6. Conclusions

NIBS might have a positive impact on attention, with no effect on executive functions. NIBS and aerobic exercise could represent a potential intervention for cognitively impaired pwMS.

Aerobic exercise was found to be associated with improved processing speed, mainly with frequency 2–3 times/week and with at least a 6-week duration. A longer study duration is needed for more consistent results. Eliminating the variability of study tools in the assessment of different cognitive domains will make analysis more feasible. Combined forms of exercise were found to be more effective in improving cognition among pwMS. Overall, exercise has a positive impact on processing speed and executive functions.

## Figures and Tables

**Table 1:** Effect of noninvasive brain stimulation on cognition.

Study/Year/Country	Design	Participant			Intervention		Location	Intensity	Duration	Tool/Outcome
		Experimental		Control	Experimental	Control				
Hsu/2023/USA [36]	parallel	19	18	20	tACS	Sham	NA	2 mA, 1 mA	20 min	SDMT improved with both 1 and 2 mA.
Simani et al./2022/Iran [37]	parallel	20		20	tDCS, CT	CT	A: F3 C: right shoulder	2 mA	30 min	IVA-2 Improved attention and inhibitory control
Gholami et al. (2021)/Iran/2021 [38]	parallel	12		12	tDCS	sham tDCS	A: F3 C: FP2	2 mA	20 min	CBS-CP improved reasoning and executive function
Seelmann-Eggebert/Germany/2021 [39]	Crossover	16			tDCS and sham 4 weeks washout		A: C3 C: supra orbital	1 mA	15 min	SDMT: no improvement
Grigorescu et al./Germany/2020 [40]	Crossover	11			tDCS or sham with 3-week washout interval		A: F3 C: F4	2 mA	20	SDMT: not improved
Fiene er al./Germany/2018 [41]	Crossover	15			tDCS with one-week washout interval		A; F3 C: right shoulder	1.5 mA	30	RT: cognitive fatigue improves
Charvet et al./USA/2018 [42]	parallel	25		20	RS tDCS, CT	CT	A: F3 C: F4	1.5 mA	20 min	BICAMS, ANT, IV Improve complex attention and response variability
Ayache et al./France/2016 [43]	Crossover	16			block of tDCS active or sham with 3 w washout		A: F3 C: Supraorbital	2 mA	20 min	SDMT not improved
Chalah et al./France/2016 [44]	Crossover	10			block tDCS active or sham with 3 w washout		A: F3 or P4 C: CZ	2 mA	20 min	ANT: not improved
Hanken et al./Germany/2016 [45]	parallel	20		20	tDCS, Vigilant task	Sham tDCS Vigilant	A: P4 C: forehead	1.5 mA	20	RT: improved
Palm/Germnay/2016 [46]	Crossover	16			blocks of tRANS followed by 3 w washout		A: F3 C: AF4	2	NA	ANT not improved
Mattioli et al./Italy/2015 [47]	parallel	10		10	tDCS CT	Sham tDCS CT	A:F3 C: Right shoulder	2	20	PASAT, WCST and SDMT improve attention and executive function

Abbreviations: IVA-2: Integrated Visual and Auditory Test; CBS-CP: Cambridge Brain Science Cognitive Platform; SDMT: Symbol–Digit Modalities Test; RT: Reaction Time; tCDS: transcranial direct current stimulation; RS tDCS: remotely supervised transcranial direct current stimulation; CT: cognitive training; BICAMS: Brief International Cognitive Assessment in MS; ANT: basic and complex attention; IV: intra-individual response variability; tRNS: transcranial random noise stimulation; transcranial alternating current stimulation (tACS); WCST: Wisconsin Card Sorting Test; PASAT Paced Auditory Serial Attention Test.

**Table 2:** Effect of different exercises on cognition among pwMS.

Study/Year/Country	Design	Participant			Intervention		Frequency	Duration	Duration Per Session	Tool/Outcome
		Exp.		Control	Experimental	Control				
Tasci/2023/Turkey [48]	RCT	18		18	Aerobic, strength	No intervention	3	12 w	NA	PBI, MFIS improved
Açık/2023/Turkey [49]	RCT	Aerobic 16	Strength 11	16	Aerobic, strength	No intervention	3	12 w	NA	BICAMS improved
Galperin//2023/USA [50]	RCT, parallel	62		62	Treadmill training with virtual reality	treadmill training	3	6 w	NA	SDMT improved
Andreu-Caravaca et al./2022/Germany [51]	RCT, parallel	18		12	Resistance	No intervention	3/W	10 w	NA	MSQOL-54 cognition improved
Bilek/2022/Turkey [52]	RCT, parallel	34		34	Aerobic and Frenkel coordination exercise	Frenkel coordination only	3 w	6 w	30 min	PASAT improved
Jeong/2021/USA [53]	RCT, parallel	29		16	Telerehabilitation system plus home-based exercise	Home-based exercise only	7/w	12 W	NA	MSQOL cognitive improved
Langeskov-Christense/2021/Denmark [54]	RCT, parallel	43		43	Supervised progressive aerobic exercise followed by self-guided physical exercise	Habitual lifestyle then supervised progressive aerobic exercise	2/w	24 w	60 min	SRT-LT SRT-C SRT-D SPART-D WLG PASAST, SDMT only improved; the rest, no effect
Savšek/2021/Slovenia [55]	RCT, parallel	14		14	Aerobic	No intervention	2	12 w	60 min	MFIS SDMT BVMT-R CVLT-II No improvement
Bckusi et al. (2020)/USA [56]	RCT, parallel	6		6	Aerobic/Functional electrical stimulation	No intervention	3/w	12 W	30 min	MSQOL-54 cognition improved
Ozdogar/2020/Turkey [57]	RCT, Parallel	Video-based, 21; conventional, 19		20	Video-based core stabilization and vs. conventional rehab vs. control group		1/w	8 W	45 min	CVLT, BVMT-R, SDMT: all are improved
Ozk §0/Turkey [19]	RCT, parallel	17		17	Aerobic and Pilates	Relaxation exercise	3/w	8 w	90 min	SRT-ST SRT-LT SDMT, SPART-T, SPART-D, PASAST, WLG Improved verbal memory, visuospatial, verbal fluency in the
Feys/2019/Belgium [58]	RCT, parallel	21		21	Aerobic	No intervention	3	12 w	NA	PASAT, WLG, SPART, SRT, DSST all improved
Baquet/2018/Germany [59]	RCT, parallel	34		34	Aerobic	No intervention	2	12 w	54.5	SDMT, PASAT, TAP, BVMT, RWT no improvement
Sebastião,/2018/USA [60]	RCT, parallel	15		10	Aerobic	No intervention	2	12 w	20 min	SDMT, CVLT, BVMT, PASAT no improvement due to small sample size
Kargarfard/2017/Iran [61]	RCT, parallel	17		15	Aerobic functional exercise and balance	No intervention	3	8 w	60 min	MFIS improved
Kjølhede/2017/Denmark [62]	RCT, parallel	17		12	Resistance	No intervention	2	24 w	55 min	MSFC improved, TUG
Sandroff/2017/USA [63]	RCT, parallel	32		30	Aerobic, balance and resistance	Stretching	3	24 w	60 min	SDMT not improved, PASAT improved
Sandroff/2016/USA [21]	RCT, parallel	4		4	Aerobic, treadmill	NA	3	12 W	20 min	CVLT-II improved, MFT, DKFFS,
Kooshiar/2015/Iran [24]	RCT, parallel	18		19	Stretching	No intervention	3	8 w	45 min	MFIS, PASAT cognitive no improved
Carter/2014/UK [64]	RCT, parallel	60		60	Aerobic	No intervention	3	6 w	60 min	MSQOL improved. PASAT.
Hoang/2016/Australia [65]	RCT, parallel	28		22	Aerobic	Regular physical therapy	2	12 w	30 min	SDMT, TMT, TUG all are improved
Oken/2004/turkey [66]	RCT, parallel	15		20	Aerobic	No intervention	1	24 w	90 min	SCWT no improvement
Oken/2004/turkey [66]	RCT, parallel	22		20	Yoga	No intervention	1	24 w	90 min	SCWT no improvement
Sandroff/2014/USA [67]	RCT	37		39	Aerobic	No intervention	NA	24 w	NA	SDMT improved
Leavitt/2014/USA [68]	RCT, parallel	1		1	Aerobic	No intervention	NA	12 W	NA	CVLT-II BVMT-R SDMT All were improved.
Briken/2014/Germany [69]	RCT progressive MS	Total: 32 Arm:10 Rowing:11 Bicycle:11		10	Arm, rowing, and bicycle	No intervention	2–3/	8–10 W	20 min	SDMT no effect LPS no improvement VLMT improved. TAP improved
Romberg/2005/Finland [70]	RCT, parallel, RRMS	47		48	Resistance	No intervention	3	24 w	NA	PASAT MSQOL-cognitive Both improved

Abbreviations: RCTs: Randomized Controlled Trials; MSQOL-54: Multiple Sclerosis Quality of Life-54; CVLT-II, California Verbal Learning; BVMT-R, Brief Visual Memory Test; SRT-LT, Selective Reminding Test—long term; SRT-ST, Selective Reminding Test—short term; SRT-C, Selective Reminding Test—consistent; SDMT, Symbol Digit Modalities Test; PASAT, Paced Auditory Serial Attention Test; WLG, Word List Generation; MFIS, Modified Fatigue Impact Scale; EXP, experimental group; CON, control group; MFIS, Modified Fatigue Impact Scale; FIS, Fatigue Impact Scale; DKEFS, Delis–Kaplan Executive Function System; MFT, modified flanker task; IC-RT, interference control of reaction time; CLTR, consistent long-term retrieval; SPART, Spatial Recall Test; SPART-D, Spatial Recall Test delayed; SPART-T, Spatial Recall Test—total; SCWT, Stroop Color–Word Interference; MSQol-54, Multiple Sclerosis Quality of Life-54; DSST, Digit Symbol Substitution Test; VLMT, Verbal Learning Memory Test; TAP, test battery for attention; RWT, Regensburg Verbal Fluency Test; TMT, Trail Making Test; TUG, timed up-and-go; PDQ, Perceived Deficit Questionnaire; PBI, Problem Solving Inventory.
